# Factors associated with 30-day readmission of patients with heart failure from a Japanese administrative database

**DOI:** 10.1186/s12872-015-0127-9

**Published:** 2015-10-24

**Authors:** Hiroki Aizawa, Shinobu Imai, Kiyohide Fushimi

**Affiliations:** Department of Health Policy and Informatics, Tokyo Medical and Dental University, Graduate School of Medicine, Tokyo, Japan; Department of Clinical Data Management and Research, Clinical Research Center, National Hospital Organization Headquarters, Tokyo, Japan

**Keywords:** Heart failure, 30-day readmission, Japanese administrative database, The Diagnosis Procedure Combination (DPC) database

## Abstract

**Background:**

Numerous studies have been conducted in many countries to identify the factors associated with readmission of patients with heart failure (HF). However, there have been no such studies utilizing a large-scale administrative database in Japan. This study aimed to establish the factors associated with 30-day readmission of patients with HF using a Japanese nationwide administrative database.

**Methods:**

Data of the index admissions of 68,257 patients discharged from 1057 participating hospitals between April 1, 2012 and March 31, 2013 were analyzed. Patients were divided into the 30-day readmission group and no readmission group according to whether unplanned HF readmission occurred within 30 days after discharge. Study variables included age, sex, New York Heart Association functional class (NYHA) at admission, Charlson Comorbidity Index (CCI), length of stay in hospital (LOS), body mass index (BMI) at admission, hospital volume reflected by the number of cases hospitalized with HF, and medical treatment at discharge.

**Results:**

The 30-day readmission and no readmission groups included 4479 and 63,778 patients, respectively. The independent factors associated with the increase in 30-day readmission were older age, higher NYHA, higher CCI, and use of the following drugs at discharge: beta blockers, loop diuretics, thiazide, and nitrates. In contrast, the independent factors associated with the decrease in 30-day readmission were longer LOS, higher BMI, and the use of angiotensin converting enzyme inhibitors (ACEs) or angiotensin II receptor blockers (ARBs), calcium channel blockers, and spironolactone.

**Conclusions:**

The results suggest that, especially during the first few weeks after discharge, careful management of HF outpatients with advanced age, high disease severity, multiple comorbidities, or taking beta blockers, loop diuretics, thiazide, and nitrates at discharge may be crucial for reducing the 30-day readmission rate.

## Background

Heart failure (HF) is a common syndrome and a frequent cause of hospital admission. About 26 million adults worldwide are living with HF, making it a global pandemic [1]. Approximately 5 million patients with HF reside in the United States, and 550,000 individuals are diagnosed for the first time every year [2]. According to the European Society of Cardiology, the prevalence of HF is 2–3 %, and there are at least 15 million patients with this disease in the 51 European countries [3]. Despite the scarcity of data, the prevalence of HF in Japan has been estimated to be approximately 1–2 %, and it is rapidly increasing [4, 5]. The causes of this trend include, among others, the increase of elderly population, higher survival rate after acute myocardial infarction, and improvements in the treatment of HF leading to better survival, such as angiotensin converting enzyme inhibitors (ACEs), angiotensin II receptor blockers (ARBs), beta blockers, aldosterone antagonists, and electrical devices [6].

The increase in the number of patients with HF led to more frequent readmissions of such individuals. Thus, recent studies reported 30-day readmission rates of approximately 25 % in the United States [7–10]. Since reduction in readmission rates might simultaneously lower the associated costs and improve the quality of care, public and private payers have progressively targeted readmissions as a focus of pay-for-performance initiatives [7, 11]. In accordance with this, basic research and hospital-driven efforts have been focused on the prediction of which patients with HF are likely to be readmitted and the design of interventions to prevent readmissions.

In worldwide numerous studies, many predictors associated with readmission of patients with HF have been recognized and racial/ethnic differences have been indicated [12, 13]. Although several registries and observational studies for patients with HF, including the Acute Decompensated Heart Failure Syndromes (ATTEND) registry [14], the Japanese Cardiac Registry of Heart Failure in Cardiology (JCARE-CARD) [15], and the Chronic Heart Failure Analysis and Registry in the Tohoku District 2 (CHART-2) Study [16], have been performed in Japan, large-scale administrative databases have not been utilized in such investigations. The aim of this study was to identify the factors associated with 30-day readmission of patients with HF in Japan, using a Japanese nationwide administrative database termed the Diagnosis Procedure Combination (DPC) database [17, 18].

## Methods

### Data source

**Fig. 1:**
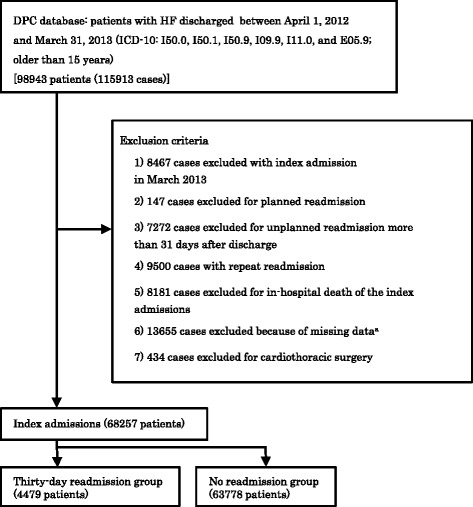
Selection of patients with heart failure from the Diagnostic Procedure Combination (DPC) database. ^a^Missing data included New York Heart Association functional class (NYHA), body mass index (BMI), and medical treatment at discharge

The present study used the DPC database from patients discharged between April 1, 2012 and March 31, 2013. The database contains both digitized discharge summaries and claims data from 1057 participating hospitals, covering approximately 48 % of all hospitalizations to acute care hospitals in Japan. The database includes patient information on demographics, primary diagnosis, comorbidities present on admission, complications occurring during hospitalization, medical procedures, medications, and materials. Patients with HF as the principal disease or the disease associated with the highest medical costs were identified based on the following codes of the 10th revision of the International Statistical Classification of Diseases (ICD10): I50.0, I50.1, I50.9, I09.9, I11.0, and E05.9. Figure 1 shows the patient selection process. This study included patients older than 15 years at the time of hospital admission. The index admissions occurred in March 2013 were excluded because we lacked the complete 30 days of follow-up. Cases with planned readmission, in-hospital death, missing data or cardiothoracic surgery were excluded. The study was approved by the Tokyo Medical and Dental University ethics committee.

### Study design

Thirty-day readmission was defined as unplanned HF readmission within 30 days after discharge (30-day readmission group). The 30-day readmission group was compared to the no readmission group, which was composed of the patients who did not require rehospitalization within the same period. If a patient was hospitalized multiple times with HF exacerbation during the study period, only the index admission of the shortest time up to rehospitalization was extracted to maintain the independence of observations. Patients who were readmitted to other hospitals were not included in the 30-day readmission group in this study, because the patient registration numbers were different for every hospital. Patients who died before readmission were also not included in the 30-day readmission group, because outpatient data after discharge from the index admission could not be surveyed.

### Variables

Variables used in the present study included age, sex, New York Heart Association functional class (NYHA) at admission, Charlson Comorbidity Index (CCI) [19, 20], length of stay in hospital (LOS), body mass index (BMI) at admission, hospital volume reflected by the number of cases hospitalized with HF, and medical treatment at discharge (ACEs or ARBs, beta blockers, calcium channel blockers, digitalis, loop diuretics, thiazide, spironolactone, and nitrates). Patients were categorized into three age groups: < 65, 65–74, and ≥ 75 years old; four CCI groups: ≤ 1, 2, 3, and ≥ 4; and three hospital volume groups: ≤ 68, 69–150, and ≥ 151 cases.

### Statistical analysis

Summary statistics of categorical patient characteristics and the details of the prescribed medicines at discharge were described as numbers and percentages, and continuous variables were described as medians and interquartile ranges (IQRs). Bi-variable analyses were employed to compare the 30-day readmission and no readmission groups for each candidate predictor using the chi-square test for categorical variables and the Wilcoxon rank-sum test for continuous variables. Multivariable logistic regression analysis after controlling simultaneously for each variable was performed to determine the factors associated with 30-day readmission of patients with HF. In multivariable regression analysis, an important assumption is that explanatory variables are independent of each other. Therefore, we used variance inflation factors (VIFs) to test for multicollinearity among the predictor variables. A VIF exceeding 10 was regarded as indicating serious multicollinearity, and a value greater than 4.0 was considered a cause for concern [21]. P-values, adjusted odds ratios (ORs), and corresponding two-sided 95 % confidence intervals (CIs) were obtained for the predictors. The statistical analyses were performed using release 11 of the JMP software package (SAS Institute Inc., Cary, NC, USA). P-values < 0.05 were considered to represent statistical significance.

## Results

This study included the index admissions of 68,257 patients who met the inclusion and exclusion criteria. Of these patients, 4479 and 63,778 were categorized into the 30-day readmission and no readmission groups, respectively.

The demographic characteristics are shown in Table 1. In unadjusted comparisons, the patients in the 30-day readmission group were significantly older (*P* < 0.0001), had higher NYHA at admission (*P* < 0.0001), higher CCI (*P* < 0.0001), and lower BMI (*P* < 0.0001) than the patients in the no readmission group. Table 2 summarizes the medications prescribed at discharge to the subjects. In unadjusted comparisons, significantly fewer patients in the 30-day readmission group were taking ACEs or ARBs (*P* < 0.0001), calcium channel blockers (*P* < 0.0001), and spironolactone (*P* < 0.0001) than the subjects in the no readmission group, whereas more patients were taking beta blockers (*P* = 0.0001), loop diuretics (*P* < 0.0001), thiazide (*P* = 0.0001), and nitrates (*P* < 0.0001).

**Table 1:** The basic characteristics of the study patients

	Readmission	No readmission	P-value
Number of patients	4479	63778	
Age (years)			<0.0001
Median	81	80	
IQR	73–86	71–86	
Category [n (%)]			
<65	487 (10.9)	9898 (15.5)	
65–74	802 (17.9)	11598 (18.2)	
≥75	3190 (71.2)	42282 (66.3)	
Male [n (%)]	2351 (52.5)	33981 (53.3)	0.305^a^
NYHA [n (%)]			<0.0001^a^
I	254 (5.7)	5411 (8.5)	
II	1235 (27.6)	19168 (30.1)	
III	1652 (36.9)	22164 (34.8)	
IV	1338 (29.9)	17035 (26.7)	
Charlson Comorbidity Index			<0.0001
Median	2	2	
IQR	1–3	1–3	
Category [n (%)]			
≤1	1181 (26.4)	19906 (31.2)	
2	1187 (26.5)	17992 (28.2)	
3	1025 (22.9)	13543 (21.2)	
≥4	1086 (24.3)	12337 (19.3)	
LOS			0.571
Median	19	19	
IQR	12–29	12–29	
BMI			<0.0001
Median	21.7	22.3	
IQR	19.2–24.4	19.7–25.2	
Hospital volume			0.914
Median	104	105	
IQR	70–147	68–150	
Category [n (%)]			
≤68	1079 (24.1)	16116 (25.3)	
69–150	2356 (52.6)	31904 (50.0)	
≥151	1044 (23.3)	15758 (24.7)	

*IQR* interquartile range; *NYHA* New York Heart Association functional class; *LOS* length of stay in hospital; *BMI* body mass index; *Hospital volume* number of cases hospitalized with heart failure

^a^Calculated using the chi-square test; the remaining P-values were calculated with the Wilcoxon rank-sum test

**Table 2:** The summary of the medications prescribed at discharge

	Readmission	No readmission	P-value
Number of patients	4479	63778	
ACEs or ARBs [n (%)]	2216 (49.5)	36076 (56.6)	<0.0001
Beta blockers [n (%)]	1883 (42.0)	24947 (39.1)	0.0001
Ca channel blockers [n (%)]	1074 (24.0)	17457 (27.4)	<0.0001
Digitalis [n (%)]	484 (10.8)	7383 (11.6)	0.119
Loop diuretics [n (%)]	3631 (81.1)	49703 (77.9)	<0.0001
Thiazide [n (%)]	375 (8.4)	4379 (6.9)	0.0001
Spironolactone [n (%)]	1383 (30.9)	22512 (35.3)	<0.0001
Nitrates [n (%)]	940 (21.0)	9850 (15.4)	<0.0001

The P-values were calculated with the chi-square test

*ACEs* angiotensin converting enzyme inhibitors; *ARBs* angiotensin II receptor blockers; *Ca channel blockers* calcium channel blockers

The VIFs for the predictor variables in this study were all < 4.0, indicating the absence of multicollinearity. The multivariable analysis revealed the following demographic factors and prescribed medications associated with increased 30-day readmission of patients with HF: older age, higher NYHA, and higher CCI, and use of beta blockers, loop diuretics, thiazide, and nitrates. In contrast, the factors associated with reduced 30-day readmission of patients with HF included longer LOS, higher BMI, and use of ACEs or ARBs, calcium channel blockers, and spironolactone (Table 3).

**Table 3:** Adjusted OR and 95 % CI for the factors associated with 30-day readmission

		Adjusted OR	95 % CI	P-value
Age	<65	reference		
	65–74	1.267	1.127–1.425	<0.0001
	≥75	1.330	1.199–1.475	<0.0001
Male		0.982	0.922–1.046	0.568
NYHA	I	reference		
	II	1.354	1.179–1.556	<0.0001
	III	1.548	1.351–1.774	<0.0001
	IV	1.627	1.416–1.869	<0.0001
CCI		1.087	1.065–1.108	<0.0001
LOS		0.997	0.995–0.998	<0.0001
BMI		0.972	0.964–0.979	<0.0001
Hospital volume		1.000	1.000–1.000	0.932
ACEs or ARBs		0.749	0.703–0.798	<0.0001
Beta blockers		1.225	1.149–1.306	<0.0001
Ca channel blockers		0.834	0.776–0.896	<0.0001
Digitalis		0.935	0.847–1.033	0.185
Loop diuretics		1.286	1.187–1.394	<0.0001
Thiazide		1.280	1.145–1.431	<0.0001
Spironolactone		0.795	0.743–0.851	<0.0001
Nitrates		1.405	1.302–1.516	<0.0001

*OR* odds ratio; *95 % CI* 95 % confidence interval; *NYHA* New York Heart Association functional class; *CCI* Charlson Comorbidity Index; *LOS* length of stay in hospital; *BMI* body mass index; *Hospital volume* number of cases hospitalized with heart failure; *ACEs* angiotensin converting enzyme inhibitors; *ARBs* angiotensin II receptor blockers; *Ca channel blockers* calcium channel blockers

## Discussion

The present study is the largest multicenter observational study using an administrative database for patients with HF in Japan. First, we identified several factors associated with the increase in 30-day readmission of patients with HF. In agreement with previous reports [22–24], these factors included older age, higher NYHA, and higher CCI. Furthermore, other factors were the use of beta blockers, loop diuretics, thiazide, and nitrates. Beta blocker therapy has contributed to reduction in mortality and long-term hospitalization in patients with systolic HF and has been used in most patients with HF [25–29]. However, initiation and up-titration of beta blockers may result in short-term hospital admission for worsening HF because of the negative inotropic and chronotropic effects. A previous study demonstrated that high starting dose of beta blockers was associated with increased readmission risk for patients with HF [30]. Although the lack of outpatient data prevented analysis of the dose of beta blockers, blood pressure, or cardiac function in the current study, we assumed that the dose of beta blockers might influence the increase in 30-day readmission. Loop diuretics remain the mainstay of decongestive therapy in acute HF and appear to benefit patients with acute HF when included in initial therapies [6]. Our result is in line with the data from a published report indicating that outpatient loop diuretics therapy was associated with increased 60-day readmission of patients with HF [31]. Thiazide is useful for reducing volume load in patients with diastolic dysfunction [32]. However, thiazide is usually utilized in combination with loop diuretics, ACEs, or ARBs, and the use of thiazide monotherapy is uncommon in patients with HF [33, 34]. Therefore, we have not been able to identify published reports of whether thiazide is associated with increased readmission of patients with HF. Nitrates have been used as vasodilators in the early stages of acute HF for many years [3, 35]. Although nitrate therapy may reduce the symptoms of dyspnea at night and during exercise in patients with HF [36], it has never been evaluated during the first few weeks after discharge in a prospective randomized study. Our results suggest that, especially during the first few weeks after discharge, careful management of HF outpatients with advanced age, high disease severity, many comorbidities, or the use of the above drugs at discharge may be an important factor for reducing 30-day readmission.

Second, the present study identified longer LOS, higher BMI, and the use of ACEs or ARBs, calcium channel blockers, and spironolactone as the factors associated with reduced 30-day readmission of patients with HF. Contrary to our finding, longer LOS has been shown to be a predictor of future readmission in Medicare analyses within the United States cohorts [12, 37]. According to a previous report, the average LOS for all hospital admissions in Japan is two to three times longer than that in the United States [38], and this difference is likely a convincing factor leading to this discrepancy. The difference in the average LOS in Japan and the United States is likely related to a number of factors, such as the social context, the universal health coverage, and the demographic cohorts. Furthermore, Japanese hospitals generally provide rehabilitation and nursing care in addition to acute medical care, which may also contribute to the longer LOS. We found that higher BMI was associated with reduced 30-day readmission of patients with HF, which seems to confirm the existence of the so-called “obesity paradox” in patients with HF discovered in previous studies [39, 40]. In contrast, a single study from Japan showed that BMI levels were not associated with rehospitalization for worsening HF [41], necessitating further research. The use of ACEs or ARBs, calcium channel blockers, and spironolactone was shown to prevent hospitalizations for HF in previous reports [42–49], and our results suggest that these factors might contribute to the decrease in 30-day readmission.

### Study limitations

Some limitations of this study should be noted. First, patients who were readmitted to other hospitals were not included in the 30-day readmission group in this study, because the patient registration numbers were different for every hospital. This point is very important and would underestimate the readmission rate. Second, our data did not include important information on the clinical status, laboratory data, and cardiac function at admission (such as blood pressure, serum creatinine levels, and ejection fraction). Consequently, the clinical characteristics of the patients hospitalized with worsening HF have not been well described. Third, because we could not combine the DPC database with outpatient claims data, outpatient data after discharge from the index admission were not surveyed. Therefore, patients who died before readmission were not included in the 30-day readmission group in this study. These points may represent disadvantages of the current study when compared with other registries and observational studies, and they may have influenced the results of our analyses.

## Conclusions

The present study is the largest Japanese multicenter observational study using an administrative database of patients with HF. According to the results of multivariable analysis, the factors associated with the increase in 30-day readmission of patients with HF were older age, higher NYHA, and higher CCI, as well as the use of beta blockers, loop diuretics, thiazide, and nitrates. In contrast, the factors associated with the decrease in 30-day readmission were longer LOS, higher BMI, and the use of ACEs or ARBs, calcium channel blockers, and spironolactone. Therefore, careful management, especially during the first few weeks after discharge, of outpatients with HF who have advanced age, high disease severity, many comorbidities, or take beta blockers, loop diuretics, thiazide, and nitrates at discharge may be crucial for reducing the 30-day readmission rate.

## Abbreviations

HF: Heart failure
NYHA: New York Heart Association functional class
CCI: Charlson comorbidity index
LOS: Length of stay in hospital
DPC: Diagnosis Procedure Combination
ACEs: Angiotensin converting enzyme inhibitors
ARBs: Angiotensin II receptor blockers
ATTEND: Acute Decompensated Heart Failure Syndromes
JCARE-CARD: Japanese Cardiac Registry of Heart Failure in Cardiology
CHART-2: Chronic Heart Failure Analysis and Registry in the Tohoku District 2
ICD10: 10th revision of the International Statistical Classification of Diseases
BMI: Body mass index
IQR: Interquartile range
VIF: Variance inflation factor
OR: Odds ratio
CI: Confidence interval
Ca channel blockers: Calcium channel blockers

## References

[1] Ambrosy AP, Fonarow GC, Butler J, Chioncel O, Greene SJ, Vaduganathan M (2014). The global health and economic burden of hospitalizations for heart failure: lessons learned from hospitalized heart failure registries. J Am Coll Cardiol.

[2] Hunt SA, Abraham WT, Chin MH, Feldman AM, Francis GS, Ganiats TG (2009). 2009 Focused update incorporated into the ACC/AHA 2005 Guidelines for the Diagnosis and Management of Heart Failure in Adults A Report of the American College of Cardiology Foundation/American Heart Association Task Force on Practice Guidelines Developed in Collaboration With the International Society for Heart and Lung Transplantation. J Am Coll Cardiol.

[3] Dickstein K, Cohen-Solal A, Filippatos G, McMurray JJ, Ponikowski P, Poole-Wilson PA (2008). ESC Guidelines for the diagnosis and treatment of acute and chronic heart failure 2008: the Task Force for the Diagnosis and Treatment of Acute and Chronic Heart Failure 2008 of the European Society of Cardiology. Developed in collaboration with the Heart Failure Association of the ESC (HFA) and endorsed by the European Society of Intensive Care Medicine (ESICM). Eur Heart J.

[4] Shiba N, Shimokawa H (2008). Chronic heart failure in Japan: implications of the CHART studies. Vasc Health Risk Manag.

[5] Okura Y, Ramadan MM, Ohno Y, Mitsuma W, Tanaka K, Ito M (2008). Impending epidemic: future projection of heart failure in Japan to the year 2055. Circ J.

[6] Gheorghiade M, Pang PS (2009). Acute heart failure syndromes. J Am Coll Cardiol.

[7] Desai AS, Stevenson LW (2012). Rehospitalization for heart failure: predict or prevent?. Circulation.

[8] Jencks SF, Williams MV, Coleman EA (2009). Rehospitalizations among patients in the Medicare fee-for-service program. N Engl J Med.

[9] Roger VL, Go AS, Lloyd-Jones DM, Adams RJ, Berry JD, Brown TM (2011). Heart disease and stroke statistics--2011 update: a report from the American Heart Association. Circulation.

[10] Nieminen MS, Brutsaert D, Dickstein K, Drexler H, Follath F, Harjola VP (2006). EuroHeart Failure Survey II (EHFS II): a survey on hospitalized acute heart failure patients: description of population. Eur Heart J.

[11] Krumholz HM, Merrill AR, Schone EM, Schreiner GC, Chen J, Bradley EH (2009). Patterns of hospital performance in acute myocardial infarction and heart failure 30-day mortality and readmission. Circ Cardiovasc Qual Outcomes.

[12] Zaya M, Phan A, Schwarz ER (2012). Predictors of re-hospitalization in patients with chronic heart failure. World J Cardiol.

[13] Vivo RP, Krim SR, Liang L, Neely M, Hernandez AF, Eapen ZJ (2014). Short- and long-term rehospitalization and mortality for heart failure in 4 racial/ethnic populations. J Am Heart Assoc.

[14] Sato N, Kajimoto K, Asai K, Mizuno M, Minami Y, Nagashima M (2010). Acute decompensated heart failure syndromes (ATTEND) registry. A prospective observational multicenter cohort study: rationale, design, and preliminary data. Am Heart J.

[15] Tsutsui H, Tsuchihashi-Makaya M, Kinugawa S, Goto D, Takeshita A (2006). Clinical characteristics and outcome of hospitalized patients with heart failure in Japan. Circ J.

[16] Shiba N, Nochioka K, Miura M, Kohno H, Shimokawa H (2011). Trend of westernization of etiology and clinical characteristics of heart failure patients in Japan--first report from the CHART-2 study. Circ J.

[17] Matsuda S (2007). Casemix as a tool for transparency of medical services. The Japanese Journal of Security Policy.

[18] Fushimi K, Hashimoto H, Imanaka Y, Kuwabara K, Horiguchi H, Ishikawa KB (2007). Functional mapping of hospitals by diagnosis-dominant case-mix analysis. BMC Health Serv Res.

[19] Charlson ME, Pompei P, Ales KL, MacKenzie CR (1987). A new method of classifying prognostic comorbidity in longitudinal studies: development and validation. J Chronic Dis.

[20] Sundararajan V, Quan H, Halfon P, Fushimi K, Luthi JC, Burnand B (2007). Cross-national comparative performance of three versions of the ICD-10 Charlson index. Med Care.

[21] Hayashi T, Boyko EJ, Leonetti DL, McNeely MJ, Newell-Morris L, Kahn SE (2004). Visceral adiposity is an independent predictor of incident hypertension in Japanese Americans. Ann Intern Med.

[22] Au AG, McAlister FA, Bakal JA, Ezekowitz J, Kaul P, van Walraven C (2012). Predicting the risk of unplanned readmission or death within 30 days of discharge after a heart failure hospitalization. Am Heart J.

[23] Islam T, O'Connell B, Lakhan P (2013). Hospital readmission among older adults with congestive heart failure. Aust Health Rev.

[24] McNaughton CD, Collins SP, Kripalani S, Rothman R, Self WH, Jenkins C (2013). Low numeracy is associated with increased odds of 30-day emergency department or hospital recidivism for patients with acute heart failure. Circ Heart Fail.

[25] The Cardiac Insufficiency Bisoprolol Study II (CIBIS-II): a randomised trial. Lancet 1999;353:9–13. 10023943

[26] Eichhorn EJ, Bristow MR (2001). The Carvedilol Prospective Randomized Cumulative Survival (COPERNICUS) trial. Curr Control Trials Cardiovasc Med.

[27] Effect of metoprolol CR/XL in chronic heart failure: Metoprolol CR/XL Randomised Intervention Trial in Congestive Heart Failure (MERIT-HF). Lancet 1999;353:2001–2007. 10376614

[28] Packer M, Coats AJ, Fowler MB, Katus HA, Krum H, Mohacsi P (2001). Effect of carvedilol on survival in severe chronic heart failure. N Engl J Med.

[29] Fonarow GC, Heywood JT, Heidenreich PA, Lopatin M, Yancy CW (2007). Temporal trends in clinical characteristics, treatments, and outcomes for heart failure hospitalizations, 2002 to 2004: findings from Acute Decompensated Heart Failure National Registry (ADHERE). Am Heart J.

[30] Allen LA, Magid DJ, Zeng C, Peterson PN, Clarke CL, Shetterly S (2012). Patterns of beta-blocker intensification in ambulatory heart failure patients and short-term association with hospitalization. BMC Cardiovasc Disord.

[31] Shah RV, McNulty S, O'Connor CM, Felker GM, Braunwald E, Givertz MM (2012). Effect of admission oral diuretic dose on response to continuous versus bolus intravenous diuretics in acute heart failure: an analysis from diuretic optimization strategies in acute heart failure. Am Heart J.

[32] Davis BR, Piller LB, Cutler JA, Furberg C, Dunn K, Franklin S (2006). Role of diuretics in the prevention of heart failure: the Antihypertensive and Lipid-Lowering Treatment to Prevent Heart Attack Trial. Circulation.

[33] Shchekochikhin D, Al Ammary F, Lindenfeld JA, Schrier R (2013). Role of diuretics and ultrafiltration in congestive heart failure. Pharmaceuticals (Basel).

[34] Ito H, Ishii K, Kihara H, Kasayuki N, Nakamura F, Shimada K (2012). Adding thiazide to a renin-angiotensin blocker improves left ventricular relaxation and improves heart failure in patients with hypertension. Hypertens Res.

[35] Metra M, Teerlink JR, Voors AA, Felker GM, Milo-Cotter O, Weatherley B (2009). Vasodilators in the treatment of acute heart failure: what we know, what we don't. Heart Fail Rev.

[36] Elkayam U, Johnson JV, Shotan A, Bokhari S, Solodky A, Canetti M (1999). Double-blind, placebo-controlled study to evaluate the effect of organic nitrates in patients with chronic heart failure treated with angiotensin-converting enzyme inhibition. Circulation.

[37] Aranda JM, Johnson JW, Conti JB (2009). Current trends in heart failure readmission rates: analysis of Medicare data. Clin Cardiol.

[38] Health at a Glance 2013. Health Care Activities: Average length of stay in hospitals. OECD. 2013. [http://www.oecd.org/els/health-systems/health-at-a-glance-2013.pdf] Accessed 11 May 2015.

[39] Fonarow GC, Srikanthan P, Costanzo MR, Cintron GB, Lopatin M (2007). An obesity paradox in acute heart failure: analysis of body mass index and inhospital mortality for 108,927 patients in the Acute Decompensated Heart Failure National Registry. Am Heart J.

[40] Zapatero A, Barba R, Gonzalez N, Losa JE, Plaza S, Canora J (2012). Influence of obesity and malnutrition on acute heart failure. Rev Esp Cardiol (Engl Ed).

[41] Hamaguchi S, Tsuchihashi-Makaya M, Kinugawa S, Goto D, Yokota T, Goto K (2010). Body mass index is an independent predictor of long-term outcomes in patients hospitalized with heart failure in Japan. Circ J.

[42] Effect of enalapril on survival in patients with reduced left ventricular ejection fractions and congestive heart failure. The SOLVD Investigators. N Engl J Med 1991;325:293**–**302. 10.1056/NEJM199108013250501 2057034

[43] Effect of enalapril on mortality and the development of heart failure in asymptomatic patients with reduced left ventricular ejection fractions. The SOLVD Investigattors. N Engl J Med 1992;327:685–691. 10.1056/NEJM199209033271003 1463530

[44] Pfeffer MA, Swedberg K, Granger CB, Held P, McMurray JJ, Michelson EL (2003). Effects of candesartan on mortality and morbidity in patients with chronic heart failure: the CHARM-Overall programme. Lancet.

[45] McMurray JJ, Ostergren J, Swedberg K, Granger CB, Held P, Michelson EL (2003). Effects of candesartan in patients with chronic heart failure and reduced left-ventricular systolic function taking angiotensin-converting-enzyme inhibitors: the CHARM-Added trial. Lancet.

[46] Granger CB, McMurray JJ, Yusuf S, Held P, Michelson EL, Olofsson B (2003). Effects of candesartan in patients with chronic heart failure and reduced left-ventricular systolic function intolerant to angiotensin-converting-enzyme inhibitors: the CHARM-Alternative trial. Lancet.

[47] Yusuf S, Pfeffer MA, Swedberg K, Granger CB, Held P, McMurray JJ (2003). Effects of candesartan in patients with chronic heart failure and preserved left-ventricular ejection fraction: the CHARM-Preserved Trial. Lancet.

[48] Packer M, O'Connor CM, Ghali JK, Pressler ML, Carson PE, Belkin RN (1996). Effect of amlodipine on morbidity and mortality in severe chronic heart failure. Prospective Randomized Amlodipine Survival Evaluation Study Group. N Engl J Med.

[49] Pitt B, Zannad F, Remme WJ, Cody R, Castaigne A, Perez A (1999). The effect of spironolactone on morbidity and mortality in patients with severe heart failure. Randomized Aldactone Evaluation Study Investigators. N Engl J Med.

